# Dimensionality and Measurement Invariance of the Italian Version of the EORTC QLQ-C30 in Postoperative Lung Cancer Patients

**DOI:** 10.3389/fpsyg.2019.02147

**Published:** 2019-10-08

**Authors:** Chiara Marzorati, Dario Monzani, Ketti Mazzocco, Francesca Pavan, Massimo Monturano, Gabriella Pravettoni

**Affiliations:** ^1^Department of Oncology and Hemato-Oncology, Faculty of Medicine and Surgery, University of Milan, Milan, Italy; ^2^Applied Research Division for Cognitive and Psychological Science, European Institute of Oncology IRCCS, Milan, Italy; ^3^Patient Safety and Risk Management Service, European Institute of Oncology IRCCS, Milan, Italy

**Keywords:** lung cancer, EORTC QLQ-C30, quality of life, validity, assessment, measurement invariance

## Abstract

**Background:**

This study aims to validate and evaluate the psychometric properties and measurement invariance of the Italian version of the European Organization for Research and Treatment of Cancer (EORTC) Quality of Life Questionnaire-Core 30 (QLQ-C30), which is a measure of quality of life (QoL) for lung cancer patients after surgery.

**Methods:**

A total of 167 lung cancer patients completed the Italian version of the EORTC QLQ-C30 questionnaire at 30 days after they received a lobectomy. The factor structure of this scale was assessed by performing confirmatory factor analysis (CFA). Measurement invariance was evaluated by considering differential item functioning (DIF) due to age, gender, and type of surgery (i.e., robot- or not robot-assisted).

**Results:**

The CFA demonstrated the validity of the factor structure of the EORTC QLQ-C30 in assessing overall health and eight distinct subscales of adverse events and functioning. Moreover, the results highlighted a minimal DIF with only trivial consequences on measurement invariance. Specifically, the DIF did not affect the mean differences of latent scores of QoL between patients undergoing robot-assisted surgery or traditional surgery.

**Conclusion:**

These findings supported the validity and suitability of the EORTC QLQ-C30 for the assessment of QoL in lung cancer patients of diverse ages and genders undergoing lobectomy with or without robot-assisted surgery.

## Introduction

Patient reported outcomes (PROs) have become important factors in cancer care to measure the patient’s perception of the health status, including treatment side effects, functional impairments, and health-related QoL (U.S. Department of Health and Human Services FDA Center for Drug Evaluation and Research et al., 2006; Mercieca-Bebber et al., 2018). Through the QoL assessment, it would be possible to obtain a more complete framework of the medical condition, especially for diseases requiring long-term care services. Among the large amount of developed instruments to evaluate patient well-being, the EORTC QLQ-C30 is the most used tool for assessing QoL in cancer-specific patients (Iravani et al., 2018). The EORTC QLQ-C30 consists of 30 self-reported questions assessing different aspects of patient functioning, global health status, and cancer-related symptoms. More specifically, it is composed of five multi-item functional scales (role, physical, cognitive, emotional, and social functioning), three multi-item symptom scales (fatigue, pain, and nausea and vomiting), individual items concerning common symptoms in cancer patients (dyspnea, insomnia, appetite loss, constipation, diarrhea, and financial difficulties), and two questions assessing overall QoL. All of the multi-item scales and single-item measures range in a score from 0 to 100, where a high score represents a higher response level. Thus, a high score for a functional scale implicates a healthy level of functioning, while a high score for a symptom scale represents a worse level of symptoms (Aaronson et al., 1993). The EORTC QLQ-C30 has been translated in over 110 languages and validated in many countries in different samples of cancer patients (Bjordal et al., 2000; EORTC Quality of Life Group, 2019). According to a cross-cultural project on a large and heterogeneous sample, the EORTC QoL Group reported robust measurement properties across various countries and languages (Scott et al., 2006, 2007). In Italy, the questionnaire has been validated only in breast and colon cancer patients (Apolone et al., 1998; Mosconi et al., 2002; Winters et al., 2014). At the same time, other authors investigated the applicability of the EORTC QLQ-C30 structure, and positively demonstrated its invariance across different cancer sites (Costa et al., 2015). Despite these psychometric properties, few scientific articles performed factor analysis for validating this tool in lung cancer patients, a clinical area in which the EORTC QLQ-C30 is the most used instrument to report patient well-being through the different phases of disease (Damm et al., 2013). To our knowledge, no published articles investigated the psychometric properties and the measurement invariance of the Italian version of the EORTC QLQ-C30 in lung cancer patients. In fact, only four studies measured QoL in Italian lung cancer patients through the administration of the EORTC QLQ-C30. Two of them were international studies and involved several countries, with all of them focusing on non-small cell lung cancer (Di Maio et al., 2004; Maione et al., 2005; Koller et al., 2017; Wood et al., 2019).

The purpose of the current study was to evaluate the factor structure proposed by Costa et al. (2015) for the EORTC QLQ-C30 in a sample of postoperative lung cancer patients who underwent lobectomy surgery. Moreover, its measurement invariance across patients of varying age, gender, and undergoing robotic or traditional surgery was also evaluated. The testing of measurement invariance is a necessary step to further evaluate any inter-individual differences.

## Materials and Methods

### Participants and Procedure

An Italian sample of 167 patients with lung cancer who were also undergoing lobectomy were recruited for the value-based project^1^ at the European Institute of Oncology in Milan between October 2015 and October 2017. Patients were included in the study if they: (1) were diagnosed with lung cancer, (2) were native Italian speakers, (3) referred to the value-based project, and (4) did not have neurological or psychopathological problems. They completed the EORTC QLQ-C30 after 30 days from surgery (Aaronson et al., 1993; Apolone et al., 1998). During the doctor’s post-operative visit, a trained nurse distributed the questionnaire to the patients and they completed it using paper and pencil. Informed consent was provided and signed by each participant. Participation in the study was voluntary and at each moment, patients could withdraw their consent. The study was developed in accordance with the principles stated in the Declaration of Helsinki (59th WMA General Assembly, Seoul, 2008) and was approved by the European Institute of Oncology Ethical Committee at the European Institute of Oncology, Milan, Italy.

### Statistical Analysis

**FIGURE 1:**
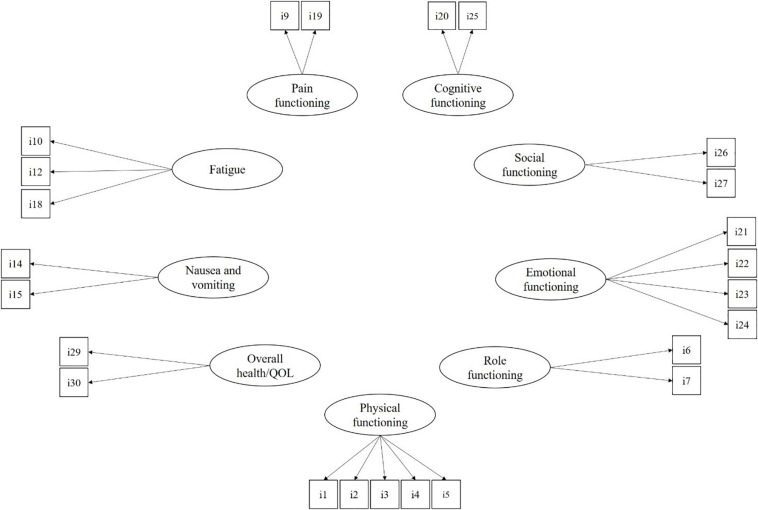
The measurement model for the EORTC QLQ-C30. For ease of interpretation, covariances among latent factors are not reported but estimated in the CFA model.

All statistical analyses were performed using the maximum likelihood with robust standard errors (MLR) estimation method with Mplus 8.2 (Muthen and Muthén, 2017). The MLR estimator is robust to strong departures from univariate and multivariate normality of observed variables. The EORTC QLQ-C30 comprises nine multiple-item dimensions and six single items. In a first step, the proposed model for the EORTC QLQ-C30 was assessed through CFA. Specifically, as reported in Figure 1, the measurement model included the nine multiple item dimensions of physical functioning (five items), role functioning (two items), emotional functioning (four items), social functioning (two items), cognitive functioning (two items), pain (two items), fatigue (three items), nausea and vomiting (two items), and overall health and QoL (two items). Following Costa et al. (2015), the six single-item dimensions (i.e., dyspnea, insomnia, appetite loss, constipation, diarrhea, and financial difficulties) were omitted from the tested model. For ease of interpretation, the covariances among latent dimensions of QoL were not reported in the figure, but they were all estimated in the analyses.

Model fit was assessed by considering five main fit indices. Specifically, a good-fitting model was indicated by a non-significant χ^2^, a RMSEA below 0.06, a SRMR <0.80, a CFI, and a TLI >0.90 (Hu and Bentler, 1999). Moreover, the 90% confidence interval for RMSEA was considered to test the null hypothesis of poor model fit. Specifically, a good-fitting model was indicated by the upper limit <0.08 and the lower limit close to zero. Finally, we considered the PCLOSE as well, a one-sided test of the null hypothesis that the model has a close fit (i.e., RMSEA equals 0.05). *P*-value >0.50 indicated a good-fitting model (Jöreskog and Sörbom, 1996).

Then, measurement invariance was evaluated by considering DIF. DIF is a prerequisite for a valid and meaningful comparison of levels of QoL across gender, age, and type of surgery (robot-assisted vs. traditional surgery). Specifically, a MIMIC was performed to assess differences in the measurement model due to age, gender, and type of surgery. A MIMIC model was performed because it has specific advantages over multiple-group CFA (MCFA) in evaluating measurement invariance. Specifically, compared to MCFA, the MIMIC model permits to: assess differences in the measurement model due to several confounding variables; simultaneously evaluate the role of dichotomous (i.e., robot-assisted vs. not robot-assisted surgery and gender) and continuous variables (i.e., age); include directly in the model continuous variables without median-splitting, mean-splitting, or subjective categorization; and test measurement invariance even with small sample size. Thus, mainly because of the low sample size, we preferred the MIMIC model over the MCFA to assess the structural invariance of the EORTC QLQ-C30. In the last decade, MIMIC model had been adopted to validly test measurement invariance of self-report measure of QoL in asthma (Mora et al., 2009) and pediatric patients (Stevanovic et al., 2016), life satisfaction (Jang et al., 2017), dispositional optimism (Steca et al., 2017), protective behavioral strategies (Treloar et al., 2014), adolescent burnout (Li et al., 2013), and depression (Skule et al., 2014).

The MIMIC model included the measurement model (i.e., the EORTC QLQ-C30 factor structure) plus a structural model assessing DIF. This structural model estimated the effect of covariates of gender, age, and type of surgery on latent dimensions of QoL and, thus, evaluated differences in these latent factors due to the three considered covariates. The structural model included the direct effects of these three covariates on items as well. In a first step, these direct effects fixed at zero. Then, modification indices were examined to ascertain whether the estimation of any of these direct effects would improve model fit. Estimation of direct effects was performed with a stepwise approach: the constraint that resulted in the greatest change of χ^2^ (i.e., highest value of the modification index) were firstly estimated. We then continued at freely estimating one direct effect at time until any modification was relevant (i.e., Δχ^2^ > 3.84). Each significant direct effect was interpreted as an indication of DIF: the likelihood to endorse an item was conditional to the specific covariate involved in the direct effect. For example, if the direct effect of age on item 1 was significant and positive, then the likelihood of endorsing this item differed between patients of different age and, specifically, younger people had lower chance to endorse this item. Thus, measurement invariance may be strongly impaired when high degree of DIF is ascertained. Age was treated as a continuous variable, whereas gender (i.e., male = 0; female = 1) and type of surgery (i.e., not robot-assisted surgery = 0; robot-assisted surgery = 1) were binary variables.

## Results

### Sample and Item Characteristics

Participants had a mean age of 66.69 ± 7.70 and 100 (59.9%) of them were males. The sample underwent lobectomy surgical procedure (*N* = 54; 32.3% with robot-assisted surgery; *N* = 113; 67.7% with not robot-assisted surgery). Other clinical variables are reported in Table 1.

**TABLE 1:** Clinical sample characteristics.

	**Sample (%)**
**ASA class**	
1	3.6
2–3	96.4
**Charlson index**	
<1	60.5
≥1	39.5
**Robot-assisted surgery**	
No	26.4
Yes	55.9
**Complications**	
No	67.1
Yes	32.9

Descriptive statistics of item response (mean, standard deviation, and minimum and maximum) are reported in Table 2.

**TABLE 2:** Descriptive statistics of item response.

**Items**	***M***	***SD***	**Min**	**Max**
i1. Do you have any trouble doing strenuous activities, like carrying a heavy shopping bag or a suitcase?	2.16	0.85	1	4
*Ha difficoltà nel fare lavori faticosi, come sollevare una borsa della spesa pesante o una valigia?*				
i2. Do you have any trouble taking a long walk?	2.171	0.87	1	4
*Ha difficoltà nel fare una lunga passeggiata?*				
i3. Do you have any trouble taking a short walk outside of the house?	1.396	0.68	1	4
*Ha difficoltà nel fare una breve passeggiata fuori casa?*				
i4. Do you need to stay in bed or a chair during the day?	1.799	0.76	1	4
*Ha bisogno di stare a letto o su una sedia durante il giorno?*				
i5. Do you need help with eating, dressing, washing yourself, or using the toilet?	1.085	0.37	1	4
*Ha bisogno di aiuto per mangiare, vestirsi, lavarsi, o andare in bagno?*				
i6. Were you limited in doing either your work or other daily activities?	1.915	0.84	1	4
*Ha avuto limitazioni nel fare il Suo lavoro o i lavori di casa?*				
i7. Were you limited in pursuing your hobbies or other leisure time activities?	1.857	0.82	1	4
*Ha avuto limitazioni nel praticare i Suoi passatempi- hobby o altre attività di divertimento o svago?*				
i9. Have you had pain?	1.883	0.85	1	4
*Ha avuto dolore?*				
i10. Did you need to rest?	2.085	0.73	1	4
*Ha avuto bisogno di riposo?*				
i12. Have you felt weak?	2.037	0.81	1	4
*Si è sentito debole?*				
i14. Have you felt nauseated	1.421	0.69	1	4
*Ha avuto un senso di nausea?*				
i15. Have you vomited?	1.049	0.29	1	4
*Ha vomitato?*				
i18. Were you tired?	2.078	0.74	1	4
*Si è sentito stanco?*				
i19. Did pain interfere with your daily activities?	1.723	0.79	1	4
*Il dolore ha interferito con le Sue attività quotidiane?*				
i20. Have you had difficulty in concentrating on things, like reading a newspaper or watching television?	1.265	0.56	1	4
*Ha avuto difficoltà a concentrarsi su cose come leggere un giornale o guardare la televisione?*				
i21. Did you feel tense?	1.719	0.74	1	4
*Si è sentito teso?*				
i22. Did you worry?	1.768	0.79	1	4
*Si è preoccupato?*				
i23. Did you feel irritable?	1.643	0.74	1	4
*Si è sentito irritabile?*				
i24. Did you feel depressed?	1.675	0.86	1	4
*Si è sentito depresso?*				
i25. Have you had difficulty remembering things?	1.394	0.63	1	4
*Ha avuto difficoltà a ricordare le cose?*				
i26. Has your physical condition or medical treatment interfered with your family life?	1.429	0.65	1	4
*Le Sue condizioni fisiche o il Suo trattamento medico hanno interferito con la Sua vita familiare?*				
i27. Has your physical condition or medical treatment interfered with your social activities?	1.582	0.73	1	4
*Le Sue condizioni fisiche o il Suo trattamento medico hanno interferito con le Sue attività sociali?*				
i29. How would you rate your overall health during the past week?	4.597	1.03	2	7
*Come valuterebbe in generale la Sua salute durante gli ultimi sette giorni?*				
i30. How would you rate your overall quality of life during the past week?	4.636	1.14	2	7
*Come valuterebbe in generale la Sua qualità di vita durante gli ultimi sette giorni?*				

### Assessing the Factor Structure

The proposed measurement model for the EORTC QLQ-C30 showed a good fit [χ^2^(216, *N* = 167) = 301.48; RMSEA = 0.05; 90% CI of RMSEA = 0.04–0.06; PCLOSE = 0.555; CFI = 0.95; TLI = 0.93; SRMR = 0.05]. The standardized loadings are displayed in Table 3. As reported, all the items had significant loadings and high loadings ranging from 0.36 to 1.08, except for i5 (λ = 0.17; SE = 0.07; *p* < 0.05) and i15 (λ = 0.13; SE = 0.12; *p* > 0.05). Specifically, while high scores of pain, fatigue, nausea and vomiting, and physical, role, cognitive, emotional, and social functioning indicated high levels of impairment, high values of overall health and QoL denoted high levels of health-related QoL.

**TABLE 3:** Standardized factors loading, standard errors, and significance for the measurement model of the EORTC QLQ-C30.

**Items**	**PF**	**RF**	**PA**	**FA**	**NV**	**CF**	**EF**	**SF**	**QL**
i1	0.63(0.06)^∗∗∗^								
i2	0.68(0.06)^∗∗∗^								
i3	0.51(0.07)^∗∗∗^								
i4	0.55(0.07)^∗∗∗^								
i5	0.17(0.07)^∗^								
i6		0.75(0.05)^∗∗∗^							
i7		0.62(0.07)^∗∗∗^							
i9			0.65(0.06)^∗∗∗^						
i19			0.75(0.06)^∗∗∗^						
i10				0.55(0.06)^∗∗∗^					
i12				0.66(0.06)^∗∗∗^					
i18				0.65(0.06)^∗∗∗^					
i14					0.40(0.17)^∗^				
i15					0.13(0.12)				
i20						0.47(0.09)^∗∗∗^			
i25						0.36(0.09)^∗∗∗^			
i21							0.60(0.06)^∗∗∗^		
i22							0.53(0.08)^∗∗∗^		
i23							0.54(0.08)^∗∗∗^		
i24							0.64(0.08)^∗∗∗^		
i26								0.51(0.08)^∗∗∗^	
i27								0.67(0.07)^∗∗∗^	
i29									0.92(0.07)^∗∗∗^
i30									1.08(0.07)^∗∗∗^

*PF, physical functioning; RF, role functioning; PA, pain; FA, fatigue; NV, nausea and vomiting; CF, cognitive functioning; EF, emotional functioning; SF, social functioning; QL, overall health and quality of life. ^∗^p < 0.05, ^∗∗^p < 0.01 and ^∗∗∗^p < 0.001.*

Table 4 displays correlations among the nine latent dimensions of QoL. Significant correlation coefficients ranged from 0.24 to 0.85 in absolute values. These correlations could be interpreted as measure of effect size of the associations among latent factors. Following suggestion by Cohen (1988), the magnitude of these coefficients was interpreted as: weak (>0.10), moderate (>0.30), and strong (>0.50). Specifically, weak associations were reported between cognitive functioning and physical functioning (*r* = 0.29), emotional functioning and nausea/vomiting (*r* = 0.29), and nausea/vomiting and health-related QoL (*r* = −0.24). A grand total of 18 correlations were large in magnitude. Physical functioning and fatigue were the latent dimensions displaying the higher number of strong correlations with other factors of QoL. Specifically, physical functioning displayed strong associations with pain (*r* = 0.54), fatigue (*r* = 0.85), health-related QoL (*r* = −0.67), role (*r* = 0.77), cognitive (*r* = 0.51), emotional (*r* = 0.50), and social functioning (*r* = 0.50). Fatigue showed strong associations with health-related QoL (*r* = −0.68), pain (*r* = 0.63), nausea/vomiting (*r* = 0.51), physical (*r* = 0.85), role (*r* = 0.76), cognitive (*r* = 0.59), emotional (*r* = 0.62), and social functioning (*r* = 0.51). Finally, role functioning was the latent dimension of QoL most strongly associated with health-related QoL (*r* = −0.72).

**TABLE 4:** Correlations (and their significance) among the nine latent dimensions of the EORTC QLQ-C30.

	**PF**	**RF**	**PA**	**FA**	**NV**	**CF**	**EF**	**SF**	**QL**
PF	–								
RF	0.77^∗∗∗^	–							
PA	0.54^∗∗∗^	0.71^∗∗∗^	–						
FA	0.85^∗∗∗^	0.76^∗∗∗^	0.63^∗∗∗^	–					
NV	0.34^∗∗∗^	0.43^∗^	0.46^∗∗∗^	0.51^∗∗∗^	–				
CF	0.51^∗∗∗^	0.48^∗∗∗^	0.29^∗∗^	0.59^∗∗∗^	0.30	–			
EF	0.50^∗∗∗^	0.52^∗∗∗^	0.45^∗∗∗^	0.62^∗∗∗^	0.29^∗^	0.41^∗∗^	–		
SF	0.50^∗∗∗^	0.54^∗∗∗^	0.37^∗∗∗^	0.51^∗∗∗^	0.08	0.50^∗∗∗^	0.46^∗∗∗^	–	
QL	–0.67^∗∗∗^	–0.72^∗∗∗^	–0.57^∗∗∗^	–0.68^∗∗∗^	−0.24^∗^	–0.51^∗∗∗^	–0.42^∗∗∗^	–0.43^∗∗∗^	–

*PF, physical functioning; RF, role functioning; PA, pain; FA, fatigue; NV, nausea and vomiting; CF, cognitive functioning; EF, emotional functioning; SF, social functioning; QL, overall health and quality of life. ^∗^p < 0.05 and ^∗∗∗^p < 0.001.*

### MIMIC Analysis of Measurement Invariance

After entering age, gender, and type of surgery in the model, goodness of fit slightly remained substantially unchanged [χ^2^(261, *N* = 167) = 385.65; RMSEA = 0.05; 90% CI of RMSEA = 0.04–0.06; PCLOSE = 0.299; CFI = 0.93; TLI = 0.91; SRMR = 0.05]. The standardize factor loadings ranged from 0.15 to 1.01. Some significant influences of the three covariates on latent factors of QoL were reported. Specifically, type of surgery was responsible for differences in nausea/vomiting (β = −0.52; SE = 0.22; *p* < 0.05), pain (β = −0.32; SE = 0.15; *p* < 0.05), and physical (β = −0.39; SE = 0.15; *p* < 0.01), role (β = −0.46; SE = 0.16; *p* < 0.01), cognitive (β = −0.31; SE = 0.15; *p* < 0.05), and social functioning (β = −0.36; SE = 0.15; *p* < 0.05).

The inspection of modification indices suggested that model fit would be improved by freely estimated the direct effect of age on item 1 (β = −0.03; SE = 0.01; *p* < 0.001). After the estimation of this effect, the model still showed a good fit [χ^2^(260, *N* = 167) = 368.42; RMSEA = 0.05; 90% CI of RMSEA = 0.04–0.06; PCLOSE = 0.491; CFI = 0.94; TLI = 0.92; SRMR = 0.05]. No other modification indices were relevant.

After controlling for this DIF, some significant influences of the three covariates on latent factors of QoL were reported. Specifically, these influences were the same as the ones reported in the previous MIMIC model (i.e., the model not freely estimating direct effects of covariates on items). Specifically, type of surgery was responsible for differences in nausea/vomiting (β = −0.52; SE = 0.22; *p* < 0.05), pain (β = −0.32; SE = 0.15; *p* < 0.05), and physical (β = −0.38; SE = 0.15; *p* < 0.01), role (β = −0.46; SE = 0.16; *p* < 0.01), cognitive (β = −0.31; SE = 0.15; *p* < 0.05), and social functioning (β = −0.36; SE = 0.15; *p* < 0.05). The only exception was that age directly influenced physical functioning (β = 0.03; SE = 0.01; *p* < 0.01). Thus, by comparing this final model with the previous one we may conclude that any bias due to DIF is only minimal and not accounting for DIF it may have only trivial consequences for the assessment of physical functioning (i.e., the magnitudes of age differences in physical functioning were comparable across the two models).

## Discussion

This study represents an evaluation of the dimensionality and measurement invariance of the Italian version of the EORTC QLQ-C30 in a sample of patients with lung cancer who underwent lobectomy surgery. Our results demonstrated the validity of the factor structure proposed by Costa et al. (2015) and thus suggested that the EORTC QLQ-C30 could be used as a valid measure of QoL in lung cancer patients undergoing lobectomy. In a previous study, Costa et al. (2015) proposed and supported this measurement model in a sample of cancer patients coming from 14 countries all over the World and considering all the types of cancer (breast, colorectal, gynecological, head and neck, lung, esophagus/stomach, and prostate cancer). Compared to a previous trial on lung cancer patients assessing the changes in QoL over time (Pompili et al., 2018), this study represents the first attempt on an Italian sample to evaluate the dimensionality and interindividual differences of patients’ QoL with different sociodemographic and clinical characteristics. Another study (Maringwa et al., 2011) analyzed previously DIF on advanced cancer patients, while the present validation article was conducted on lung cancer patients with a primitive diagnosis.

The questionnaire comprises nine different dimensions. While one factor assesses “overall health and health-related QoL,” the remaining eight factors measure distinct symptoms and functioning, namely nausea/vomiting, pain, fatigue for the symptoms’ subscales, and physical, role, emotional, cognitive, and social functioning for the functioning subscales. All the nine subscales were significantly and strongly loaded by their relative items. The only exception was the nausea and vomiting dimension: one out of its two items exhibited a non-significant and very low loading on its factor. Further research is needed to better assess the validity of this subscale in evaluating symptoms of nausea and vomiting in lung cancer patients and, if necessary, to develop more reliable items to evaluate this kind of adverse events.

Moreover, this study is the first one to evaluate the psychometric properties of the Italian version of the EORTC QLQ-C30 in lung cancer patients and assess its measurement invariance and DIF due to age, gender, and robot-assisted versus not robot-assisted surgery. The presence of measurement invariance is one of the necessary steps in efficient and reliable evaluation of interindividual differences in QoL within samples of lung cancer patients and it represents a prerequisite to validly compare levels of overall health across patients of different genders and genders undergoing lobectomy with or without robot-assisted surgery.

Our main results attested that only one item displayed a trivial DIF. Specifically, compared to younger patients, the elderly were more likely to endorse Item 1 (i.e., “Do you have any trouble doing strenuous activities, like carrying a heavy shopping bag or a suitcase?”) on a four-point scale (i.e., 1 = “Not at all”; 2 = “A little”; 3 = “Quite a bit”; and 4 = “Very much”). However, the magnitude of this DIF was very small.

Finally, the last step in the evaluation of DIF involved the assessment of mean differences of nine latent scores of QoL across patients of different gender, varying age, and underwent robot-assisted or traditional surgery. The main aim of this analysis was to ascertain whether not controlling for DIF may lead to consequences for the assessment of QoL (i.e., mean differences in QoL differ when controlling or not controlling or DIF). These results highlighted that the DIF had only an irrelevant effect on the estimation of differences in latent means of QoL among patients. Accidentally, the results coming from this last step also highlighted that younger patients displayed higher levels of physical functioning than elderly ones and that robot-assisted surgery may promote better QoL 1 month after surgery. Specifically, compared to patients undergoing traditional surgery, people treated with robot-assisted surgery displayed lower pain, nausea and vomiting, as well as better physical, role, cognitive, and social functioning. This latter result is consistent with empirical evidence showing that lung cancer patients treated with robotic thoracic surgery reported a reduced postoperative pain and complications, fewer functional impairments, and a lower need of blood transfusions (Cheng et al., 2007; Nasir et al., 2014). However, it’s noteworthy that the main aim of this analysis was to assess the magnitude and the influence of DIF on mean differences of the nine latent scores of QoL; we did not aim at assessing differences due to age, gender, and type of surgery on patients’ QoL. Moreover, since we did not balance the baseline characteristics (i.e., QoL itself) between patient underwent robot-assisted or traditional surgery, these results may not be interpreted in a casual way.

Current results may be considered in light of some main limitations. Specifically, it was not possible to test convergent and/or divergent validity of the EORTC QLQ-C30 due to a lack of other self-report measures of patients’ well-being. Nevertheless, a previous Italian validation of the questionnaire reported a substantial convergent validity (Apolone et al., 1998), even though not in lung cancer patients. Finally, these statistical analyses must be taken with caution due to the relatively small sample size. Specifically, as highlighted by Kline (2015), the median of typical sample sizes in structural equation modeling studies is about 200 cases. Thus, our sample size of 167 lung cancer patients is slightly below this common standard. However, lower sample sizes are commonly recruited when the specific population being studied is restricted in size and it is difficult to reach higher sample sizes (Kline, 2015). Thus, while the low sample size may represent a limit of our study, this size is a direct consequence of our target population. Because of this small sample, structural invariance of the EORTC QLQ-C30 was assessed by performing MIMIC model and DIF analysis which, compared to MCFA, permit to better test measurement invariance even with small sample size. Future research collecting larger samples would be needed to further assess the factor structure of the EORTC QLQ-C30 in lung cancer patients underwent lobectomy with or without robot-assisted surgery.

Despite these limitations, our findings attested the goodness of the nine-factor structure of the Italian version of the EORTC QLQ-C30 in lung cancer patients and its measurement invariance in assessing QoL in patients with varying ages genders undergoing lobectomy with or without robot-assisted surgery. This is also the first study validating a QoL questionnaire on lung cancer patients. In fact, other scales have not been already validated among Italian lung cancer samples. Additionally, the EORTC QLQ-C30 assesses more dimensions related to a cancer diagnosis than other questionnaires. As a practical consequence, we advise that nine distinct scores of overall health, pain, fatigue, nausea/vomiting, physical, social, role, emotional, and cognitive functioning should be computed for evaluating lung cancer patients’ QoL in future research and clinical practice. The valid and reliable assessment of adverse events and functioning in lung cancer patients is a relevant and prognostic factor in patient’s recovery. In fact, patient survival is highly affected by treatment side-effects such as fatigue, loss of appetite, dyspnea, and coughing, as well as physical, psychological, cognitive, and social functioning (Efficace et al., 2006; Braun et al., 2011; Polanski et al., 2016). The EORTC QLQ-C30 may help healthcare stakeholders in measuring and monitoring QoL in both clinical and research fields. In particular, QoL in lung cancer has been studied to understand patients’ health status during processes aimed to stop smoking and how it may influence patients’ preferences in medical decision-making. It was also used to better investigate possible long-term effects of rumination on patients’ recovery and well-being (Gorini et al., 2018; Masiero et al., 2019). In a patient-centered approach, the measurement of QoL would be also important to assess how individual differences and cognitive processes may influence patient well-being in different medical conditions (Pravettoni and Gorini, 2011; Cutica et al., 2014; Arnaboldi et al., 2015).

## Conclusion

In conclusion, the EORTC QLQ-C30 is a useful and valid self-report tool and it can be used to assess interindividual differences of QoL in lung cancer patients in both clinical and research contexts.

## Data Availability Statement

The datasets generated for this study are available on request to the corresponding author.

## Ethics Statement

The studies involving human participants were reviewed and approved by the European Institute of Oncology IRCCS, Milan, Italy. The patients/participants provided their written informed consent to participate in this study.

## Author Contributions

KM, CM, MM, FP, and GP: concept and design. FP and CM: acquisition of the data. CM and DM: statistical analysis, interpretation of the data, and manuscript writing. CM, DM, KM, and GP: drafting revision of the manuscript for important intellectual content. MM and GP: supervision. All authors read and approved the final manuscript.

## Conflict of Interest

The authors declare that the research was conducted in the absence of any commercial or financial relationships that could be construed as a potential conflict of interest.

## Footnotes

1 The value-based project is a multidisciplinary project where medical, economical, and psychological outcomes are collected through standardized questionnaires at different time points consistently with patient’s recovery and follow ups. The aim of this project is to create predictive models of care pathways of the major cancer diagnosis.

## Abbreviations

CFA: confirmatory factor analysis
CFI: comparative fit index
DIF: differential item functioning
EORTC QLQ-C30: European Organization for Research and Treatment of Cancer (EORTC) Quality of Life Questionnaire-Core 30 (QLQ-C30)
MCFA: multiple-group factor analysis
MIMIC: multiple indicators–multiple causes
MLR: robust maximum likelihood
PCLOSE: probability of close fit
QoL: quality of life
RMSEA: root mean square error of approximation
SRMR: standardized root mean square residual
TLI: tucker–lewis index.
